# Lambda Red recombinase-mediated integration of the high molecular weight DNA into the *Escherichia coli* chromosome

**DOI:** 10.1186/s12934-016-0571-y

**Published:** 2016-10-05

**Authors:** Mario Juhas, James W. Ajioka

**Affiliations:** Department of Pathology, University of Cambridge, Tennis Court Road, Cambridge, CB2 1QP UK

**Keywords:** Synthetic biology, *Escherichia coli*, Lambda Red recombineering, Chromosomal integration, High molecular weight DNA

## Abstract

**Background:**

*Escherichia coli* K-12 is a frequently used host for a number of synthetic biology and biotechnology applications and chassis for the development of the minimal cell factories. Novel approaches for integrating high molecular weight DNA into the *E. coli* chromosome would therefore greatly facilitate engineering efforts in this bacterium.

**Results:**

We developed a reliable and flexible lambda Red recombinase-based system, which utilizes overlapping DNA fragments for integration of the high molecular weight DNA into the *E. coli* chromosome. Our chromosomal integration strategy can be used to integrate high molecular weight DNA of variable length into any non-essential locus in the *E. coli* chromosome. Using this approach we integrated 15 kb DNA encoding sucrose catabolism and lactose metabolism and transport operons into the *fliK* locus of the flagellar region 3b in the *E. coli* K12 MG1655 chromosome. Furthermore, with this system we integrated 50 kb of *Bacillus subtilis* 168 DNA into two target sites in the *E. coli* K12 MG1655 chromosome. The chromosomal integrations into the *fliK* locus occurred with high efficiency, inhibited motility, and did not have a negative effect on the growth of *E. coli*.

**Conclusions:**

In addition to the rational design of synthetic biology devices, our high molecular weight DNA chromosomal integration system will facilitate metabolic and genome-scale engineering of *E. coli.*

**Electronic supplementary material:**

The online version of this article (doi:10.1186/s12934-016-0571-y) contains supplementary material, which is available to authorized users.

## Background

*Escherichia coli* K-12 is a gram-negative model bacterium used as a host for a number of synthetic biology and biotechnology applications [1–3]. Furthermore, *E. coli* is considered to be among the most promising chassis for the development of the minimal cell factories [4–7]. Novel approaches for introducing synthetic DNA, particularly the high molecular weight DNA, into *E. coli* would therefore greatly facilitate engineering efforts in this bacterium.

Integration of synthetic DNA into the *E. coli* chromosome mitigates against many problems associated with the maintenance of DNA on plasmids or bacterial artificial chromosomes (BACs) [8–11]. Chromosomal integration avoids complications arising from issues such as plasmid segregation or plasmid-maintenance associated metabolic burden. Furthermore, maintenance of plasmids and BACs in the cell requires constant antibiotic selection pressure [8–11]. Recent *E. coli* chromosomal integration approaches include the bacteriophage integrase- mediated recombination between phage attachment (*att*) sites dubbed clonetegration [12] and the bacteriophage lambda Red recombinase- mediated recombination employing yeast homing mitochondrial I-SceI endonuclease [13, 14], knock-in/knock-out (KIKO) vectors [15] and plasmid pSB1K3(FRTK) [16].

The chromosomal integration approaches differ in their flexibility, speed and the size of the DNA fragments they can integrate into the chromosome. While there had been studies describing high molecular weight DNA integration into the chromosomes of some bacteria, such as *Bacillus subtilis* [17–19], this was not reported in *E. coli* yet. The currently used lambda Red recombination methods relying on a selection with a single antibiotic gene flanked by FLP recombinase target (FRT) sites are time-consuming due to the necessity to flip-out the antibiotic resistance gene in order to use it again. Furthermore, the traditional lambda Red recombineering employing antibiotic cassette flanked by FRT sites leaves a single FRT site in the chromosome after flipping-out of the antibiotics gene [20]. The undesired recombination between the two FRT sites (first on the introduced DNA fragment and second in the chromosome) makes the repeated use of the cassettes flanked with FRT sites unsuitable for integration of large DNA sequences into the same target locus. Plasmid pSB1K3(FRTK), clonetegration, KIKO-vectors, and yeast homing mitochondrial I-SceI endonuclease-based methods were shown to successfully integrate up to 10 kb DNA fragments into the *E. coli* chromosome [12–15].

Here, we developed a reliable and flexible lambda Red recombinase-based approach that utilizes overlapping DNA fragments for integration of the high molecular weight DNA of virtually any length into any non-essential target site in the *E. coli* chromosome. We demonstrate the utility of this method by integrating 15 kb DNA encoding sucrose and lactose catabolism pathways and 50 kb of *B. subtilis* 168 DNA into the *E. coli* K12 MG1655 chromosome.

## Results and discussion

### *fliK* of the flagellar region 3b as the chromosomal integration target site

The potential integration target sites in the *E. coli* chromosome vary in their suitability for integration of synthetic DNA [16]. Genes involved in the biogenesis and regulation of the flagellum are considered to be particularly suitable target sites for integration of genetic circuits due to their high expression, good characterization and non-essentiality for *E. coli*. Flagellar genes are situated in the genomic regions frequently occupied by DNA-binding proteins (highly expressed extended protein occupancy domains, heEPODs) [21, 22]. Furthermore, unlike the traditionally used phage attachment (*att*) sites, flagellar genes are present and well-conserved in all commonly used industrial *E. coli* strains, such as MG1655, DH10B, BL21-DE3, and W3110 [4, 21, 22, 25].

Here, we chose to integrate high molecular weight DNA into the *fliK* locus of the *E. coli* K12 MG1655 flagellar region 3b. We selected the *fliK* locus as the integration target site because our previous analyses of the *E. coli* K12 MG1655 flagellar regions 1, 2, 3a, and 3b revealed that the flagellar region 3b supports the highest integration efficiency of all flagellar regions [16, 23, 24]. Furthermore, *fliK* was shown to support the highest integration efficiency from the *E. coli* K12 MG1655 flagellar region 3b.

**Fig. 1:**
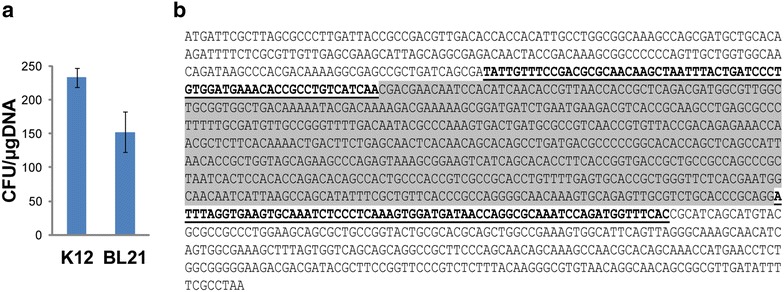
The *fliK* locus as the target for chromosomal integration of DNA. **a** Figure shows integration efficiency of the kanamycin resistance cassette (approximately 2 kb) into the *fliK* locus in the *E. coli* strains K12 MG1655 (K12) and BL21-DE3 (BL21). Means and standard deviations from three experiments are shown. **b** The sequence of the *fliK* locus of the *E. coli* chromosome, which was used as the target site for the chromosomal integrations. The figure shows the whole sequence of the *fliK* open reading frame. Integration primer parts are *highlighted bold* and *underlined*. The *fliK* sequence which was deleted from the engineered strains as a result of DNA integration by lambda Red-mediated homologous recombination is *highlighted grey*

Some commonly used industrial *E. coli* strains, such as BL21-DE3, are often causing problems by genomic engineering. This is usually due to the missing *att* sites used in many chromosomal integration approaches. Unlike *att* sites, the *fliK* locus in the strain BL21-DE3 has 100 percent homology with the strain K12 MG1655. To further verify the suitability of the *fliK* locus for integration of synthetic DNA, we compared the integration efficiency in strains BL21-DE3 and K12 MG1655. The efficiency of integration of the kanamycin resistance cassette (approximately 2 kb) into the *fliK* locus of the strain BL21-DE3 was high and comparable to that observed in the strain K12 MG1655 (Fig. 1a). This confirms the suitability of the *fliK* locus for integration of the synthetic DNA across variable *E. coli* strains. The exact position within the *fliK* open reading frame where the integrations occured is shown in Fig. 1b.

### Strategy of the high molecular weight DNA integration into the *E. coli* chromosome

**Fig. 2:**
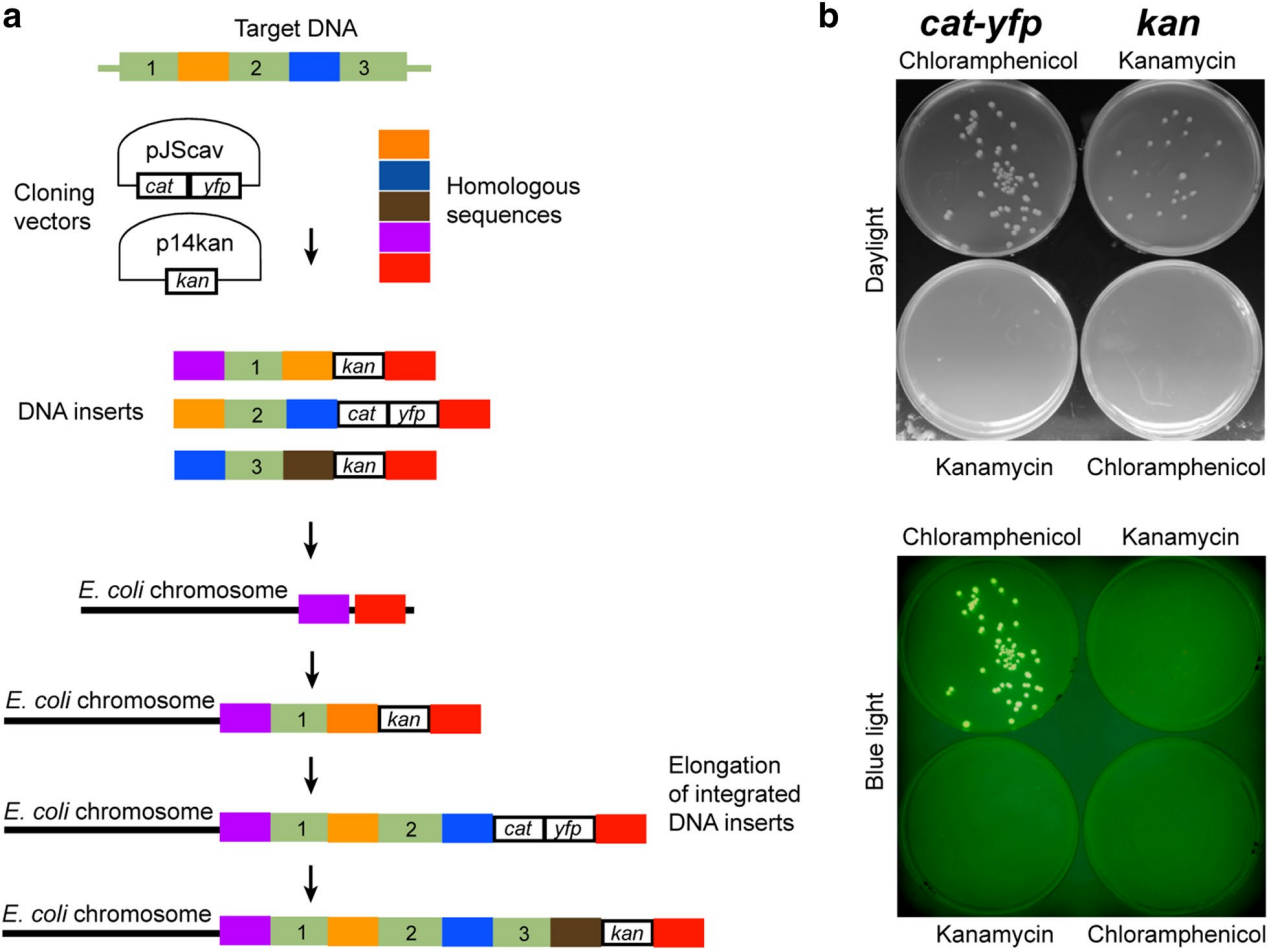
Strategy of the high molecular weight DNA integration into the *E. coli* chromosome. **a** Figure depicts main steps of the integration of the high molecular weight DNA into the *E. coli* chromosome. DNA fragments to be integrated into the *E. coli* chromosome are first tagged with the selectable markers by cloning into plasmids p14kan and pJScav. p14kan and pJScav encode the kanamycin cassette (*kan*) and chloramphenicol-yellow fluorescent protein cassette (*cat*-*yfp*), respectively. DNA fragments attached to *kan* or *cat*-*yfp* cassettes are transformed into the electro-competent *E. coli* cells harbouring plasmid pKM208. pKM208- encoded lambda Red recombination system integrates DNA fragments into the chromosome by homologous recombination. Successful transformants from the chromosomal integration of the first DNA fragment attached to *kan* cassette are selected on medium with kanamycin. Second fragment attached to *cat*-*yfp* cassette integrates into the chromosome next to the first fragment, thus replacing the *kan* resistance marker. Alternative use of *kan* and *cat*-*yfp* cassettes allows elongation of the integrated DNA sequence. **b** Growth and antibiotic selection of *E. coli* harbouring integrated high molecular weight DNA tagged with *kan* or *cat*-*yfp* cassette. Strains with *kan* and *cat*-*yfp* cassettes grow only on kanamycin and chloramphenicol plates, respectively. Furthermore, cells with *cat*-*yfp* cassette produce fluorescent light

Our strategy for the integration of the high molecular weight DNA molecules into the *E. coli* chromosome is outlined in Fig. 2. This approach utilizes the Red recombinase system of the bacteriophage lambda for integration of DNA fragments into the *E. coli* chromosome via recombination between homologous sequences [16]. Phage-based systems are a useful tool for genome engineering as they increase the otherwise very low rate of homologous recombination in some bacteria, such as *E. coli* [13, 15, 20, 25]. The lambda Red recombinase system employed in our approach is composed of three proteins Bet, Exo and Gam. Bet and Exo bind to the ends of the introduced DNA fragment and facilitate homologous recombination and integration into the chromosome. This is possible due to Gam-facilitated inhibition of the host RecBCD exonuclease V which, when active, degrades foreign DNA [20]. Lambda Red recombinase system in our approach is introduced into the host *E. coli* cells on plasmid pKM208. The expression of the lambda Red recombinase system from the lacZ promoter is tightly controlled by the presence or absence of Isopropyl β-d-1-thiogalactopyranoside (IPTG) in the growth medium [26].

DNA fragments to be integrated into the *E. coli* chromosome are first cloned into the plasmids p14kan and pJScav next to the kanamycin cassette (*kan*) and chloramphenicol-yellow fluorescent protein cassette (*cat*-*yfp*), respectively (Fig. 2a). Cloning of DNA fragments into p14kan and pJScav is facilitated by Gibson isothermal assembly approach [27, 28]. Next, DNA fragments are PCR amplified together with the attached *kan* or *cat*-*yfp* cassettes and sequences homologous to the target region in the chromosome. Sequences homologous to the target region in the *E. coli* chromosome (at least 60 bp in length) are introduced into the PCR amplified DNA fragments on primers. The length of the homology regions can be further increased by designing primers which generate longer overlapping regions between the DNA fragments for increased integration efficiency.

DNA fragments tagged with *kan* or *cat*-*yfp* cassettes are transformed into the electro-competent *E. coli* cells harbouring plasmid pKM208 with lambda Red recombination system. The pKM208- encoded lambda Red recombination system facilitates integration of DNA fragments into the *E. coli* chromosome (Fig. 2a). If the first DNA fragment is tagged with *kan* cassette, successful recombinants are selected by growing on kanamycin (Fig. 2b). The integration of the following DNA fragment tagged with *cat*-*yfp* cassette results in replacement of the *kan* resistance marker with *cat*-*yfp* cassette and successful recombinants are easily detectable by growth on chloramphenicol and yellow fluorescent protein (mVenus) expression (Fig. 2b). Elongation of the integrated DNA sequence is facilitated by the alternative use of *kan* and *cat*-*yfp* cassettes (Fig. 2a).

### Integration of the sucrose and lactose catabolism pathways into the *E. coli* chromosome

To demonstrate the utility of our system we first integrated sucrose and lactose catabolism pathways into the *E. coli* strain K12 MG1655 (Ec*Δlac*) chromosome. *E. coli* K12 MG1655 can not normally utilize sucrose. We obtained DNA encoding the sucrose catabolism pathway from *E. coli* W, which is the only non-pathogenic *E. coli* strain that can utilize sucrose as a carbon source [15, 29].

**Fig. 3:**
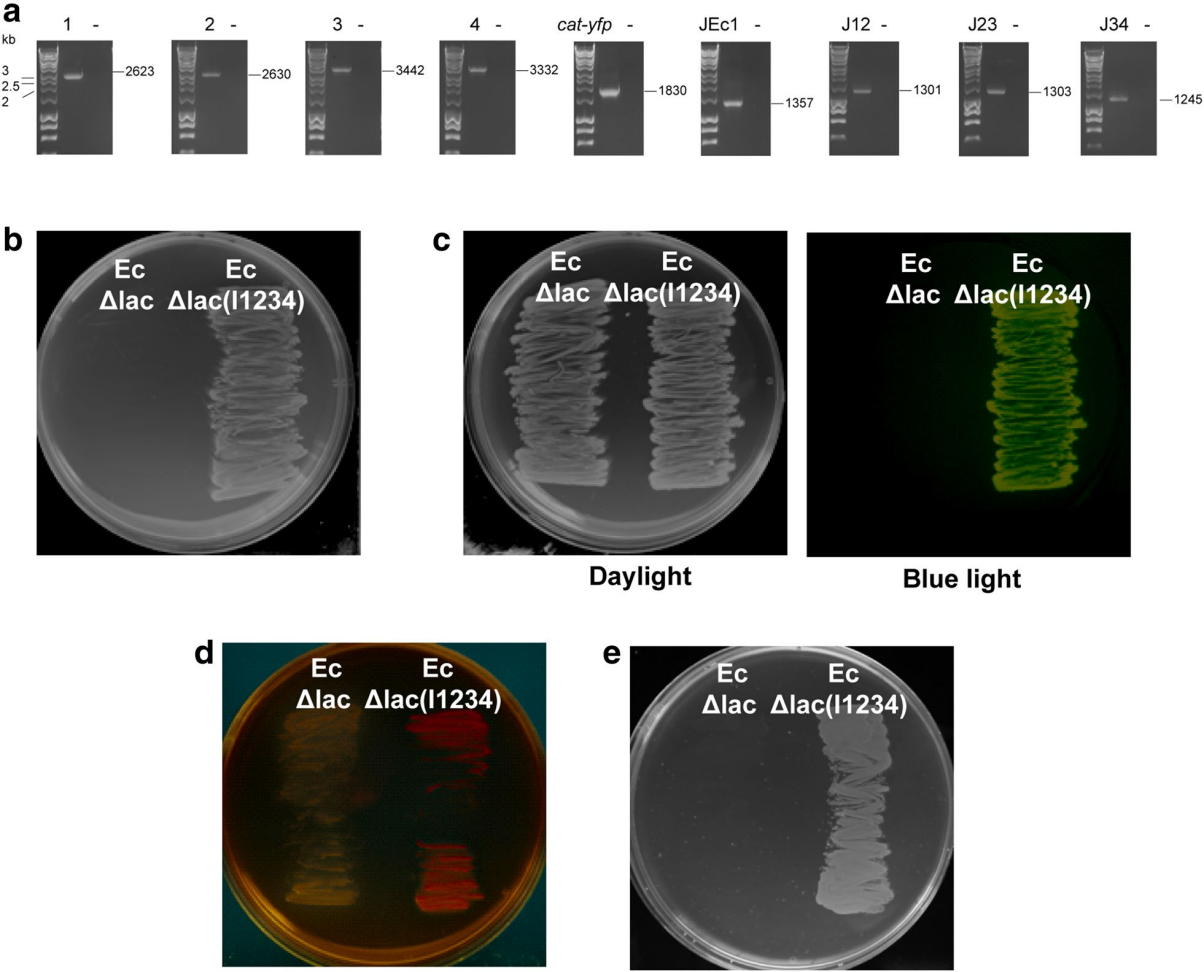
Chromosomal integration of sucrose and lactose catabolism pathways into the *E. coli* chromosome. **a** PCR confirmation of the integration of the four DNA fragments and *cat*-*yfp* cassette (15 kb DNA) into the *fliK* locus of the *E. coli* (Ec*Δlac*) chromosome. 1,2,3,4,*cat*-*yfp*: DNA fragments 1,2,3,4 and *cat*-*yfp* cassette integrated into Ec*Δlac*(I1234); negative control (Ec*Δlac*); JEc1, J12, J23, J34: junctions between the chromosome and DNA fragment 1, DNA fragments 1 and 2, DNA fragments 2 and 3, DNA fragments 3 and 4 in Ec*Δlac*(I1234), respectively. Expected amplicon sizes are shown on the *right*. **b** LB agar with chloramphenicol was used to examine chloramphenicol resistance in Ec*Δlac* (*left*) and Ec*Δlac*(I1234) (*right*). Only the engineered strain Ec*Δlac*(I1234) grew on chloramphenicol. **c** Ec*Δlac* (*left*) and Ec*Δlac*(I1234) (*right*) grown on LB plates were investigated for the expression of the integrated yellow fluorescent protein (mVenus) using *blue light*. Ec*Δlac*(I1234) emitted *yellow* fluorescent light. **d** MacConkey agar was used to analyze the ability to ferment lactose in Ec*Δlac* (*left*) and Ec*Δlac*(I1234) (*right*). The red color of Ec*Δlac*(I1234) is caused by a pH change resulting from lactose fermentation. **e** M9 medium supplemented with sucrose (20 mg/ml) was used to investigate the ability to utilize sucrose as a carbon source in Ec*Δlac* (*left*) and Ec*Δlac*(I1234) (*right*). Ec*Δlac*(I1234) grew on the minimal sucrose medium

Four DNA fragments (each approximatelly 3 kb in length) were first cloned into plasmids p14kan and pJScav for tagging with *kan* or *cat*-*yfp* cassettes (Fig. 2a). The first DNA fragment tagged with *kan* cassette was PCR amplified using primers harbouring sequences homologous to the *fliK* locus in the Ec*Δlac* chromosome (Additional file 1) and electroporated into the host *E. coli* with pKM208-encoded lambda Red recombination system. The successful transformants were selected on plates with kanamycin (Fig. 2b). The second DNA fragment tagged with *cat*-*yfp* cassette was PCR amplified using forward primer with sequence homologous to the *fliK* locus and reverse primer with homology to the sequence of the first DNA fragment already integrated into the chromosome (Additional file 1). Electroporation of the second fragment into the host *E. coli* with pKM208-encoded lambda Red recombination system led to its integration into the chromosome and replacement of the *kan* resistance marker with *cat*-*yfp* cassette. Successful recombinants grew on plates with chloramphenicol and were easily detectable by the yellow fluorescent protein (mVenus) expression (Fig. 2b). The alternative use of *kan* and *cat*-*yfp* cassettes led to the integration of all four DNA fragments into the *E. coli* chromosome. The integrated DNA has length of approximately 15 kb and encodes the whole sucrose catabolism and lactose metabolism and transport pathways, in addition to yellow fluorescent protein (mVenus) and chloramphenicol resistance-encoding open reading frames (Additional file 2). The integrations into the *E. coli* chromosome were verified by diagnostic PCR with flanking primers (Fig. 3a; Additional file 1) and sequencing. PCR amplifications and sequencing across junctions showed that the DNA fragments were integrated into the *E. coli* chromosome in the correct order (Fig. 3a; Additional file 1). Furthermore, the chromosomal integration of the yellow fluorescent protein (mVenus), chloramphenicol resistance, sucrose and lactose catabolism pathways was confirmed phenotypically (Fig. 3b–e). The engineered strain Ec*Δlac*(I1234) grew on chloramphenicol (Fig. 3b), emitted yellow fluorescent light (Fig. 3c) and fermented lactose (Fig. 3d) and sucrose (Fig. 3e).

### Integration of the 50 kb *B. subtilis* DNA into the *E. coli* chromosome

We obtained high molecular weight DNA for cloning into the *E. coli* K12 MG1655 chromosome from *B. subtilis* 168. We chose *B. subtilis* due to high DNA sequence variability between *B. subtilis* and *E. coli* to avoid undesired recombination between the host and the donor DNA. Furthermore, the *B. subtilis* 168 DNA region chosen does not harbour any known operons whose expression could be detrimental to the host *E. coli* (Additional file 3).

**Fig. 4:**
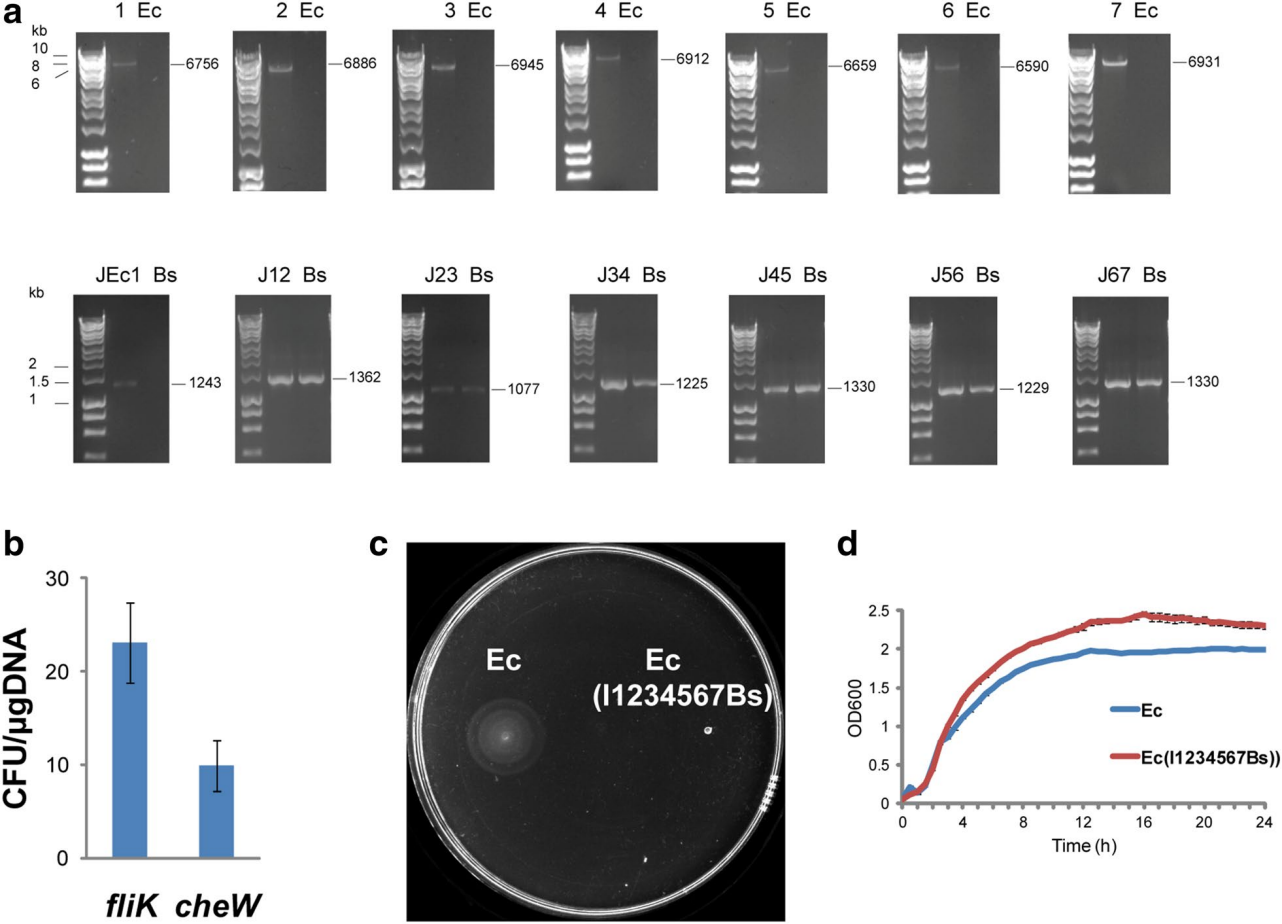
Chromosomal integration of 50 kb DNA into *E. coli*. **a** PCR verification of the integration of the seven DNA fragments (50 kb DNA length in total) into the *E. coli* K12 MG1655 chromosome. *Ec E. coli* K12 MG1655 wild type; *Bs B. subtilis* 168 wild type; 1,2,3,4,5,6,7: DNA fragments 1,2,3,4,5,6,7 in Ec(I1234567Bs); JEc1, J12, J23, J34, J45, J56, J67: junctions between the *E. coli* chromosome and DNA fragment 1, DNA fragments 1 and 2, DNA fragments 2 and 3, DNA fragments 3 and 4, DNA fragments 4 and 5, DNA fragments 5 and 6, DNA fragments 6 and 7 in Ec(I1234567Bs), respectively. Expected amplicon sizes are shown on the* right*. **b** Efficiency of integration of the 50 kb *B. subtilis* DNA into the *fliK* and *cheW* loci in the *E. coli* K12 MG1655 chromosome. Integration efficiencies were calculated from the number of colony forming units per µg of electroporated DNA. The *figure* shows means and standard deviations calculated from integration of seven DNA fragments. **c** Motility of the *E. coli* K12 MG1655 wild type (Ec) and strain Ec(I1234567Bs) harboring integration of the 50 kb *B. subtilis* DNA in the *fliK* locus of the flagellar region 3b. Overnight *E. coli* cultures (2 μl, OD_600_ of 1.0) were inoculated on the motility agar plates and analyzed after 5 h of growth at 37 °C. **d** Growth rate of the *E. coli* K12 MG1655 wild type (Ec) and strain Ec(I1234567Bs) harboring integration of the 50 kb *B. subtilis* DNA in the *fliK* locus of the flagellar region 3b measured with the microplate reader (Fluostar Omega). The means and standard errors were calculated from three independent experiments

Seven DNA fragments (each approximatelly 6–7 kb in length) were first cloned into p14kan and pJScav (Fig. 2a). The first PCR amplified DNA fragment tagged with *kan* cassette and harbouring sequences homologous to the *fliK* locus (Additional file 1) was electroporated into host *E. coli* with pKM208-encoded lambda Red recombination system. The successful transformants were selected on plates with kanamycin (Fig. 2b). The integration of the next DNA fragment tagged with *cat*-*yfp* cassette was detected by yellow fluorescent protein (mVenus) expression and growth on chloramphenicol (Fig. 2b). The alternative use of *kan* and *cat*-*yfp* cassettes led to the integration of all seven DNA fragments (with total length of approximately 50 kb) into the *E. coli* K12MG1655 chromosome. The integrations into the *E. coli* K12 MG1655 chromosome were verified by diagnostic PCR with flanking primers (Fig. 4a; Additional file 1) and sequencing. PCR amplifications and sequencing across junctions showed that the DNA fragments were integrated into the *E. coli* K12 MG1655 chromosome in the correct order (Fig. 4a).

To assess the suitability of the *fliK* locus for integration of the high molecular weight DNA, we compared the integration efficiency of the *fliK* locus with another randomly selected integration target site in the *E. coli* chromosome, *cheW* (Fig. 4b). Integration efficiencies were determined from the number of colony forming units per microgram of DNA. This analysis showed that the *fliK* locus supports integration of the high molecular weight *B. subtilis* DNA with higher efficiency than the *cheW* locus (Fig. 4b).

Genes encoding flagellar functions are important for the cell’s motility. To verify that integration of the high molecular weight DNA into the *fliK* locus of *E. coli* K12 MG1655 impacts host cell’s motility, we analyzed the engineered strain Ec(I1234567Bs) on the motility agar plate (Fig. 4c). The motility the strain Ec(I1234567Bs) was completely eliminated (Fig. 4c). Integrations of the high molecular weight DNA into the *E. coli* chromosome should not have a negative effect on the host cell’s growth. To test the effect of chromosomal integration of the high molecular weight DNA on the growth rate, we used the engineered strains Ec(I1Bs), Ec(I12Bs), Ec(I123Bs), Ec(I1234Bs), Ec(I12235Bs), Ec(I123456Bs), and Ec(I1234567Bs) harbouring integrated DNA of increasing length (up to 50 kb). The cell growth was quantified by measuring absorbance of the bacterial cultures with the microplate reader (Fluostar Omega). The absorbance of the *E. coli* K12 MG1655 wild type was compared with that of the engineered strains Ec(I1Bs), Ec(I12Bs), Ec(I123Bs), Ec(I1234Bs), Ec(I12235Bs), Ec(I123456Bs), and Ec(I1234567Bs) grown at 37 °C for 24 h (Fig. 4d; Additional file 4). Integration of *B. subtilis* DNA fragments of various lengths into the *fliK* locus of the *E. coli* chromosome produced viable transformants whose growth was not impaired when compared with the wild type *E. coli* K12 MG1655. The strains Ec(I1Bs), Ec(I12Bs), Ec(I123Bs), Ec(I1234Bs), Ec(I12235Bs), Ec(I123456Bs), and Ec(I1234567Bs) harbouring integrated DNA grew slightly better than the wild type *E. coli* K12 MG1655 (Fig. 4d; Additional file 4). Better growth of the strains with integrations into the *fliK* locus could be due to the lower metabolic burden to the cell resulting from the loss of the flagellar function. The *fliK* knockout strain grew only slightly better than the wild type *E. coli* K12 MG1655 (Additional file 4). This corroborates our previous findings, where similar growth pattern was observed for other mutants in the flagellum-encoding genes [16, 23, 24]. Good growth is a desirable feature for metabolic engineering and synthetic biology applications to generate more efficient and productive microbial cell factories.

The comparison of the integration efficiencies with another chromosomal integration target site, unimpaired growth and eliminated motility of the host cells show that the *fliK* locus of *E. coli* K12 MG1655 is suitable for chromosomal integration of the high molecular weight DNA.

## Conclusions

We set out to provide the synthetic biology community with the reliable system for the integration of the high molecular weight DNA into the *E. coli* chromosome. To this end we developed a lambda Red recombinase-based method which utilizes overlapping DNA sequences for chromosomal integration of the high molecular weight DNA. Our chromosomal integration strategy is more flexible and allows integration of larger DNA molecules into the *E. coli* chromosome than the previous methods. Furthermore, the alternative use of *kan* and *cat*-*yfp* cassettes allows faster chromosomal integration process than the standard lambda Red recombineering methods, which rely on curing-out the lambda Red recombinase-encoding vector from the cell, before introducing second, FLP recombinase-encoding plasmid [20]. The length of the DNA fragments integrated into the *E. coli* chromosome by previous lambda Red recombinase-based approaches was about 10 kb [12–16]. The chromosomal integration strategy presented here can be used to integrate high molecular weight DNA of virtually any length into the *E. coli* chromosome. Furthermore, it does not target only a few integration sites, but allows DNA integration into any non-essential locus. Using this approach we integrated 15 kb DNA encoding sucrose and lactose catabolism pathways into the *fliK* locus of the flagellar region 3b and 50 kb *B. subtilis* DNA into two target sites in the *E. coli* K12 MG1655 chromosome. We showed that integration of 50 kb of the foreign DNA into the *fliK* locus inhibits motility of *E. coli*, but does not have a negative effect on the growth. Our chromosomal integration system will facilitate rational design of synthetic biology devices in *E. coli*. By allowing integration of the high molecular weight DNA, including whole operons and metabolic pathways into the *E. coli* chromosome, it can be used for metabolic and genome-scale engineering of this bacterium. Furthermore, it can be easily adapted for use in other bacteria with the functional lambda Red recombination system.

## Methods

### Bacterial strains and growth conditions

Bacterial strains and plasmids used in this study are listed in Table 1. Luria-Bertani medium (LB) was used for the routine growth of bacteria. Bacteria cultivated in the liquid LB were grown on a rotatory shaker at 200 rpm and 37, 30 or 42 °C, depending on the requirements of the experiments. Bacteria cultivated on LB agar (10 mg/ml) plates were grown at 37, 30 or 42 °C for 24 h. When necessary, LB medium was supplemented with kanamycin (50 µg/ml), ampicillin (100 µg/ml) or chloramphenicol (30 µg/ml) to grow *E. coli* and kanamycin (5 µg/ml) or chloramphenicol (5 µg/ml) to cultivate *B. subtilis.* Lactose fermentation experiments were performed using MacConkey agar (Sigma). The ability to utilize sucrose was investigated using M9 medium supplemented with thiamine (1 µg/ml) and sucrose (20 mg/ml) [15].

**Table 1:** Bacterial strains and plasmids used in this study

	Characteristics	Reference
Strains		
Ec	*E. coli* wild type strain K12 MG1655	[34]
Bs	*B. subtilis* Marburg wild type 168, *trpC2*	[35]
BL21	*E. coli* strain BL21-DE3	CGSC stock
Ec*ΔfliK*	*E. coli* K12 MG1655 with deleten *fliK*	This study
Ec*Δlac*	*E. coli* K12 MG1655 with deleted *lac* operon	[14]
Ec(I1Bs)	Ec with *B. subtilis* DNA fragment 1 integrated	This study
Ec(I12Bs)	Ec with *B. subtilis* DNA fragments 1,2 integrated	This study
Ec(I123Bs)	Ec with *B. subtilis* DNA fragments 1,2,3 integrated	This study
Ec(I1234Bs)	Ec with *B. subtilis* DNA fragments 1,2,3,4 integrated	This study
Ec(I12345Bs)	Ec with *B. subtilis* DNA fragments 1,2,3,4,5 integrated	This study
Ec(I123456Bs)	Ec with *B. subtilis* DNA fragments 1,2,3,4,5,6 integrated	This study
Ec(I1234567Bs)	Ec with *B. subtilis* DNA fragments 1,2,3,4,5,6,7 integrated	This study
Ec*Δlac*(I1)	Ec*Δlac* with DNA fragment 1 integrated	This study
Ec*Δlac*(I12)	Ec*Δlac* with DNA fragments 1,2 integrated	This study
Ec*Δlac*(I123)	Ec*Δlac* with DNA fragments 1,2,3 integrated	This study
Ec*Δlac*(I1234)	Ec*Δlac* with DNA fragments 1,2,3,4 integrated	This study
Plasmids		
pJScav	*cat-yfp* cassette	[36]
p14kan	*kan* cassette	[16]
pKM208	plasmid with lambda Red system, IPTG inducible	[26]
pCP20	plasmid encoding flippase (FLP) recombinase	[20]
p14kan(I1Bs)	p14kan with *B. subtilis* DNA fragment 1	This study
pJScav(I2Bs)	pJScav with *B. subtilis* DNA fragment 2	This study
p14kan(I3Bs)	p14kan with *B. subtilis* DNA fragment 3	This study
pJScav(I4Bs)	pJScav with *B. subtilis* DNA fragment 4	This study
p14kan(I5Bs)	p14kan with *B. subtilis* DNA fragment 5	This study
pJScav(I6Bs)	pJScav with *B. subtilis* DNA fragment 6	This study
p14kan(I7Bs)	p14kan with *B. subtilis* DNA fragment 7	This study
p14kan(I1)	p14kan with DNA fragment 1	This study
pJScav(I2)	pJScav with DNA fragment 2	This study
p14kan(I3)	p14kan with DNA fragment 3	This study
pJScav(I4)	pJScav with DNA fragment 4	This study

### DNA integration into the *E. coli* chromosome

Integrations of DNA fragments into the *E. coli* chromosome were performed employing previously described method [16]. Briefly, DNA fragments were first PCR amplified using primers harboring flanking sequences homologous to the target region in the *E. coli* K12 MG1655 chromosome (at least 60 bp in length) and purified by gel extraction. As longer sequences greatly improve integration efficiency, the length of the homology regions can be further increased by designing primers generating longer overlapping regions between the DNA fragments. Plasmid pKM208 encoding lambda Red recombination system was introduced into *E. coli* K12 MG1655 by electroporation and successful transformants were selected on ampicillin at 30 °C. Overnight *E. coli* K12 MG1655 culture harboring pKM208 was inoculated into a fresh liquid LB medium (1:100 dilution) and grown to OD_600_ of 0.2 at 30 °C. Then, 1 mM IPTG was added and bacteria were grown to the final OD_600_ of 0.4–0.6. Bacteria were washed twice with glycerol (100 µl/ml) and resuspended in glycerol (100 µl/ml) (100 µl per 100 ml culture). Bacterial cells were then transformed with the gel purified DNA fragments by electroporation. In our settting, the best transformation efficiency was achieved when using 100–150 µl of the PCR product (approximatelly 5 µg of DNA). This volume was electroporated into 100 µl of the freshly prepared electro-competent cells harbouring pKM208. Transformants harboring the chromosomaly integrated DNA fragments were selected on kanamycin or chloramphenicol plates at 37 °C. Successful chromosomal integrations were verified by diagnostic PCR with flanking primers and sequencing. In general, approximately 50 % of the transformants harbored correctly integrated DNA fragment. The cells were transformed newly with pKM208 after each round of integration to increase chromosomal integration efficiency. After the last round of integration the temperature sensitive plasmid pKM208 can be cured out by growing transformants at 42 °C. Chemically competent bacteria were prepared by the modified Hanahan method [30]. Electro-competent *E. coli* were prepared by the modified Miller and Nickoloff method [31]. The whole process for integration of a single DNA fragment into the *E. coli* chromosome using this system takes about 4 days. This is faster than the standard lambda Red recombineering methods which necessitate curing-out the lambda Red recombinase system- encoding vector from the cell, prior to introducing another, FLP recombinase-encoding, plasmid. On the first day, DNA fragment is cloned into pJScav or p14kan by Gibson Assembly and transformed into competent *E. coli* strain. On the second day, correctly assembled plasmids are identified by colony PCR, correctly assembled colonies are inoculated for plasmid preparation and the recipient *E. coli* harbouring pKM208-encoded lambda Red recombination system is inoculated into LB with ampicillin. On the third day, plasmids pJScav/p14kan with assembled DNA are isolated and sequenced, DNA fragments tagged with *cat*-*yfp* or *kan* cassettes are PCR amplified and transformed into induced competent cells with pKM208. On the fourth day, the integrated DNA fragments are identified by colony PCR and quality controlled by sequencing and diagnostic PCR. The chromosomal integration of multiple DNA fragments can be speeded up by editing several fragments simultaneously (e.g. cloning of all fragments into pJScav and p14kan can be performed the same day).

### DNA amplification and modification methodology

Dream Taq master mix kit (Thermo Scientific) or Phusion DNA polymerase (Thermo Scientific) were used to amplify DNA fragments, according to the protocol provided by the supplier. PCR amplifications of DNA fragments were routinely performed in 50 µl reaction volumes. Oligonucleotide primers for PCR amplifications were synthesized by Sigma-Aldrich and Integrated DNA Technologies (IDT). DNA fragments were routinely assembled in a 5.2 µl reaction volume [16] employing Gibson isothermal assembly [27, 28]. Successful assemblies of DNA fragments were confirmed using diagnostic PCR with flanking primers and sequencing. Sequencing was performed by Source Bioscience (Cambridge, UK). DNA fragments were routinely purified by extraction from the agarose (10 mg/ml) gel using Qiaquick Gel Extraction kit (Qiagen), according to the manufacturer’s instructions. Plasmids were isolated with Qiaprep Spin Miniprep kit (Qiagen). *B. subtilis* genomic DNA was isolated using GeneJET genomic DNA purification kit (Thermo Scientific), according to the supplier’s instructions.

### Microplate reader measurement of the absorbance

Growth rate of bacterial strains was determined with the microplate reader (Fluostar Omega, BMG Labtech, UK). Briefly, overnight liquid cultures of the analyzed bacterial strains were diluted in a fresh LB medium to starting OD_600_ of 0.05 and 200 µl of these cultures were transferred into the wells of the 96 well microplates (flat-bottomed, clear, Sterilin Sero-Well, UK). The microplates were cultivated in the microplate reader at 37 °C for 24 h. Absorbance was determined with the automatically repeated protocol using following parameters: double orbital shaking at 500 r.p.m, absorbance filter 600 nm, cycle time: 30 min. The mean and standard errors were determined from three independent biological replicates.

### Motility assessment

The motility of the bacterial strains was evaluated using the motility agar plates. The motility agar plates were prepared by pouring 100 ml of motility agar [tryptone (100 mg/ml), NaCl (50 mg/ml), Bacto-Agar (2.5 mg/ml) (Difco)] in the 13 cm plates and let to set overnight. 2 μl of the overnight bacterial cultures diluted to OD_600_ of 1.0 were inoculated in the middle of the motility plates pre-warmed to 37 °C. Plates were incubated for 4–6 h at 37 °C prior to taking the pictures.

### Databases and sequence analyses

The *B. subtilis* genome sequence was obtained from the SubtiWiki (http://subtiwiki.uni-goettingen.de/) and BioCyc (http://bsubcyc.org/) databases. The *E. coli* K-12 MG1655 genome sequence was obtained from the *E. coli* K-12 project website (http://www.xbase.ac.uk/genome/escherichia-coli-str-k-12-substr-mg1655). The sequences of the standard DNA parts were obtained from the Registry of Standard Biological Parts (http://parts.igem.org/Main_Page). Sequencing was performed by Source Bioscience (Cambridge, UK). Sequences were compared employing TBLASTX, BLASTN [32], and position-specific iterated BLAST (PSI-BLAST) [33] from the National Center for Biotechnology Information (NCBI) website (http://ncbi.nlm.nih.gov).

## Additional files

10.1186/s12934-016-0571-y Primers used in this study.

10.1186/s12934-016-0571-y Integrated sucrose and lactose catabolism pathways. Figure shows open reading frames of the 15 kb DNA encoding sucrose catabolism and lactose metabolism and transport pathways integrated into the *fliK* locus of the *E. coli* K12 MG1655 (Ec*Δlac*) chromosome. Csc: sucrose catabolism genes; Lac: lactose metabolism and transport genes; mVenus: yellow fluorescent protein; Cat: chloramphenicol.

10.1186/s12934-016-0571-y Integrated *B. subtilis* DNA. Figure shows open reading frames of the high molecular weight DNA from *B. subtilis* 168 integrated into the *E. coli* K12 MG1655 chromosome. Hypotheticals with unknown function are highlighted black. Oxred: oxidoreductase; Atransf: Acetyl transferase; Cys: cystathionine metabolism; Man: mannose metabolism; Sp: sporulation; PdaC: peptidoglycan metabolism; ThiS, ThaS: thiamine metabolism.

10.1186/s12934-016-0571-y Growth rates. Figure shows growth rates of the engineered strains Ec(I1Bs), Ec(I12Bs), Ec(I123Bs), Ec(I1234Bs), Ec(I12235Bs), Ec(I123456Bs), Ec(I1234567Bs) and strain Ec*ΔfliK* compared to *E. coli* K12 MG1655 wild type (Ec) measured with the microplate reader (Fluostar Omega). The means and standard errors were calculated from three biological replicates. The integration into the *fliK* locus did not have a negative effect on the growth of the host *E. coli*.

## Abbreviations

BAC: bacterial artificial chromosome
*cat*-*yfp*: chloramphenicol-yellow fluorescent protein cassette
heEPODs: highly expressed extended protein occupancy domains
IPTG: isopropyl β-d-1-thiogalactopyranoside
*kan*: kanamycin cassette
KIKO vector: knock-in/knock-out vector
LB medium: Luria-Bertani medium
FLP recombinase: flippase recombinase
FRT site: FLP recombinase target site
