# Supplementary material for: Lambda Red recombinase-mediated integration of the high molecular weight DNA into the Escherichia coli chromosome
Source: Microb Cell Fact. 2016 Oct 5;15:172. doi: 10.1186/s12934-016-0571-y (PMC5050610; doi:10.1186/s12934-016-0571-y)
Supplement: Supplementary file 1 — 10.1186/s12934-016-0571-y Primers used in this study. [file 12934_2016_571_MOESM1_ESM.doc]

**Supporting Table 1. Primers used in this study.**

**Primer (Sequence 5’→ 3’)**

p14-I1Bs-iF: p14kan(I1Bs) Gibson Isothermal Assembly forward

AACTGCAGGTCGACGGATCCACATGTACTGCTGAATTTAA

p14-I1Bs-iR: p14kan(I1Bs) Gibson Isothermal Assembly reverse

GTGACACCTTGCCCTTTTTTGTCTCCTCATTTACAGCTCC

p14-I1Bs-vF: p14kan(I1Bs) Gibson Isothermal Assembly forward

GGAGCTGTAAATGAGGAGACAAAAAAGGGCAAGGTGTCAC

p14-I1Bs-vR: p14kan(I1Bs) Gibson Isothermal Assembly reverse

TTAAATTCAGCAGTACATGTGGATCCGTCGACCTGCAGTT

pJS-I2Bs-iF: pJScav(I2Bs) Gibson Isothermal Assembly forward

TAACCTGCCCCGTTAGTTGAAGGTATATGCTCAAAAAGCA

pJS-I2Bs-iR: pJScav(I2Bs) Gibson Isothermal Assembly reverse

CGCCAGCAACGCGGCCCGACTTTATTGTTCACAAAAAAG

pJS-I2Bs-vF: pJScav(I2Bs) Gibson Isothermal Assembly forward

CTTTTTTGTGAACAATAAAGTCGGGCCGCGTTGCTGGCG

pJS-I2Bs-vR: pJScav(I2Bs) Gibson Isothermal Assembly reverse

TGCTTTTTGAGCATATACCTTCAACTAACGGGGCAGGTTA

p14-I3Bs-iF: p14kan(I3Bs) Gibson Isothermal Assembly forward

AACTGCAGGTCGACGGATCCCTGAATGGATAACTGGACA

p14-I3Bs-iR: p14kan(I3Bs) Gibson Isothermal Assembly reverse

GTGACACCTTGCCCTTTTTTGCCGAGAAACAGTATCCCG

p14-I3Bs-vF: p14kan(I3Bs) Gibson Isothermal Assembly forward

CGGGATACTGTTTCTCGGCAAAAAAGGGCAAGGTGTCAC

p14-I3Bs-vR: p14kan(I3Bs) Gibson Isothermal Assembly reverse

TGTCCAGTTATCCATTCAGGGATCCGTCGACCTGCAGTT

pJS-I4Bs-iF: pJScav(I4Bs) Gibson Isothermal Assembly forward

TAACCTGCCCCGTTAGTTGACCAGTCGGAATCATAGCTTA

pJS-I4Bs-iR: pJScav(I4Bs) Gibson Isothermal Assembly reverse

CGCCAGCAACGCGGCCCGACGGAAAAGTATATGTAACG

pJS-I4Bs-vF: pJScav(I4Bs) Gibson Isothermal Assembly forward

CGTTACATATACTTTTCCGTCGGGCCGCGTTGCTGGCG

pJS-I4Bs-vR: pJScav(I4Bs) Gibson Isothermal Assembly reverse

TAAGCTATGATTCCGACTGGTCAACTAACGGGGCAGGTTA

p14-I5Bs-iF: p14kan(I5Bs) Gibson Isothermal Assembly forward

AACTGCAGGTCGACGGATCCGGTGATACTGATTCGTGATT

p14-I5Bs-iR: p14kan(I5Bs) Gibson Isothermal Assembly reverse

GTGACACCTTGCCCTTTTTTGATGCATGGCTGAAGTGACG

p14-I5Bs-vF: p14kan(I5Bs) Gibson Isothermal Assembly forward

CGTCACTTCAGCCATGCATCAAAAAAGGGCAAGGTGTCAC

p14-I5Bs-vR: p14kan(I5Bs) Gibson Isothermal Assembly reverse

AATCACGAATCAGTATCACCGGATCCGTCGACCTGCAGTT

pJS-I6Bs-iF: pJScav(I6Bs) Gibson Isothermal Assembly forward

TAACCTGCCCCGTTAGTTGAGCAAACACTTAAGGTATCGC

pJS-I6Bs-iR: pJScav(I6Bs) Gibson Isothermal Assembly reverse

CGCCAGCAACGCGGCCCGAAAATTGACGAACTTGAAAAT

pJS-I6Bs-vF: pJScav(I6Bs) Gibson Isothermal Assembly forward

ATTTTCAAGTTCGTCAATTTTCGGGCCGCGTTGCTGGCG

pJS-I6Bs-vR: pJScav(I6Bs) Gibson Isothermal Assembly reverse

GCGATACCTTAAGTGTTTGCTCAACTAACGGGGCAGGTTA

p14-I7Bs-iF: p14kan(I7Bs) Gibson Isothermal Assembly forward

AACTGCAGGTCGACGGATCCCCATGTGTTTGAAACGGATT

p14-I7Bs-iR: p14kan(I7Bs) Gibson Isothermal Assembly reverse

GTGACACCTTGCCCTTTTTTGCAGTTGAAGAACAGTGTCC

p14-I7Bs-vF: p14kan(I7Bs) Gibson Isothermal Assembly forward

GGACACTGTTCTTCAACTGCAAAAAAGGGCAAGGTGTCAC

p14-I7Bs-vR: p14kan(I7Bs) Gibson Isothermal Assembly reverse

AATCCGTTTCAAACACATGGGGATCCGTCGACCTGCAGTT

I1357Bs-int-F: I1Bs, I3Bs, I5Bs, I7Bs integration primer forward

TATTGTTTCCGACGCGCAACAAGCTAATTTACTGATCCCTGTGGATGAAACACCGCCTGTCATCAAGTATCACGAGGCAGAATTTCAGAT

I246Bs-int-F: I2Bs, I4Bs, I6Bs integration primer forward

TATTGTTTCCGACGCGCAACAAGCTAATTTACTGATCCCTGTGGATGAAACACCGCCTGTCATCAAGCCGCCGGGCGTTTTTTAT

I1Bs-int-R: I1Bs integration primer reverse

GTGAAACCATCTGGATTTGCGCCTGGTTATCATCCACTTTGAGGGAGATTTGCACTTCACCTAAATCGGTTTTAAAGAAAAAGGGCAGG

I2Bs-int-R: I2Bs integration primer reverse

CGCGGAAACCCATGCCTACGATGGAAAAAGCCCTTATTACAATG

I3Bs-int-R: I3Bs integration primer reverse

GCGAACATGATAATCTAGGCTCACAAAAAACCTTGGAGAAGTTAGGG

I4Bs-int-R: I4Bs integration primer reverse

GTCCCCCTCTAAAAAGAGAAGGGACCTTATTTTCGCTTCAAG

I5Bs-int-R: I5Bs integration primer reverse

GATGCATGGCTGAAGTGACGGTCTTGGACTGGCTATTCTC

I6Bs-int-R: I6Bs integration primer reverse

GTCAGAGCATATCGTCGTGTATGACGGTGAAAAGCCTGTCGGTG

I7Bs-int-R: I7Bs integration primer reverse

CAGTGTCCGACTGGCTGCTATACCAACCTTTTAAACCCTACG

I2-int-R: I2 integration primer reverse

GCTTAACCAGGATGATTAAAATGACGCAATCTCGATTGCATGCGG

I4-int-R: I4 integration primer reverse

GGAAGCAAAACACCAGCAGCAGTTTTTCCAGTTCCGTTTATCCG

fliKint-iF: fliK integration primer forward

ATGCTGCACAAGATTTTCTCGCGTTGTTGAGCGAAGCATTAGCAGGCGAGACAACTACCGACAAATATCACGAGGCAGAATTTCAGAT

fliKint-iR: fliK integration primer reverse

TTAGGCGAAAATATCAACGCCGCTGTTGCCTGTTACACGCCCTTGTAAAGAGACGGGAACCGGAAGTTTTAAAGAAAAAGGGCAG

lacint-iF: lac integration primer forward

TTAAACTGACGATTCAACTTTATAATCTTTGAAATAATAGTGCTTATCCCGGTCGTTTATTTCGCGGTACCACGAGGCAGAATTTCAGAT

lacint-iR: lac integration primer reverse

CACCATCGAATGGCGCAAAACCTTTCGCGGTATGGCATGATAGCGCCCGGAAGAGAGTCAATTCAGCGGTTTTAAAGAAAAAGGGCAG

p14-I1-iF: p14kan(I1) Gibson Isothermal Assembly forward

AACTGCAGGTCGACGGATCCGGATGATGTTGATAAAACG

p14-I1-vR: p14kan(I1) Gibson Isothermal Assembly reverse

CGTTTTATCAACATCATCCGGATCCGTCGACCTGCAGTT

p14-I1-vF: p14kan(I1) Gibson Isothermal Assembly forward

GCCTGTACCTTCAGCAATATAGAAAAAAAGGGCAAGGTGTCAC

p14-I1-iR: p14kan(I1) Gibson Isothermal Assembly reverse

GTGACACCTTGCCCTTTTTTTCTATATTGCTGAAGGTACAGGC

pJS-I2-iF: pJScav(I2) Gibson Isothermal Assembly forward

TAACCTGCCCCGTTAGTTGAATGGCTTCATTAAAGGATGT

pJS-I2-vR: pJScav(I2) Gibson Isothermal Assembly reverse

ACATCCTTTAATGAAGCCATTCAACTAACGGGGCAGGTTA

pJS-I2-vF: pJScav(I2) Gibson Isothermal Assembly forward

GTTTATCACTATGCGTAACTTCGGGCCGCGTTGCTGGCG

pJS-I2-iR: pJScav(I2) Gibson Isothermal Assembly reverse

CGCCAGCAACGCGGCCCGAAGTTACGCATAGTGATAAAC

p14-I3-iF: p14kan(I3) Gibson Isothermal Assembly forward

AACTGCAGGTCGACGGATCCAATTTCGCTGGTGGTCAGAT

p14-I3-vR: p14kan(I3) Gibson Isothermal Assembly reverse

ATCTGACCACCAGCGAAATTGGATCCGTCGACCTGCAGTT

p14-I3-vF: p14kan(I3) Gibson Isothermal Assembly forward

TTTTGCGCCATTCGATGGTAAAAAAGGGCAAGGTGTCAC

p14-I3-iR: p14kan(I3) Gibson Isothermal Assembly reverse

GTGACACCTTGCCCTTTTTTACCATCGAATGGCGCAAAA

pJS-I4-iF: pJScav(I4) Gibson Isothermal Assembly forward

TTAAACTGACGATTCAACTTTATAATCTAACCTGCCCCGTTAGTTGA

pJS-I4-vR: pJScav(I4) Gibson Isothermal Assembly reverse

TCAACTAACGGGGCAGGTTAGATTATAAAGTTGAATCGTCAGTTTAA

pJS-I4-vF: pJScav(I4) Gibson Isothermal Assembly forward

TTTTCATCATATTTAATCAGTCGGGCCGCGTTGCTGGCG

pJS-I4-iR: pJScav(I4) Gibson Isothermal Assembly reverse

GCCAGCAACGCGGCCCGACTGATTAAATATGATGAAAA
