# Supplementary figures and images for: Lambda Red recombinase-mediated integration of the high molecular weight DNA into the Escherichia coli chromosome
Source: Microb Cell Fact. 2016 Oct 5;15:172. doi: 10.1186/s12934-016-0571-y (PMC5050610; doi:10.1186/s12934-016-0571-y)

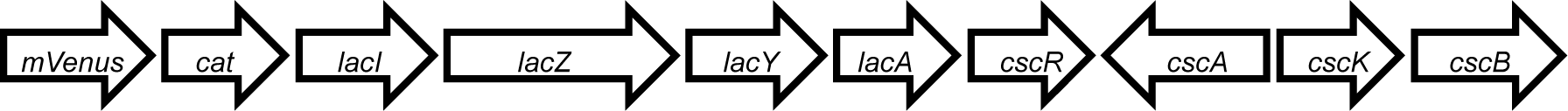

Supplement: Supplementary file 2 — 10.1186/s12934-016-0571-y Integrated sucrose and lactose catabolism pathways. Figure shows open reading frames of the 15 kb DNA encoding sucrose catabolism and lactose metabolism and transport pathways integrated into the fliK locus of the E. coli K12 MG1655 (EcΔlac) chromosome. Csc: sucrose catabolism genes; Lac: lactose metabolism and transport genes; mVenus: yellow fluorescent protein; Cat: chloramphenicol. [file 12934_2016_571_MOESM2_ESM.tif]

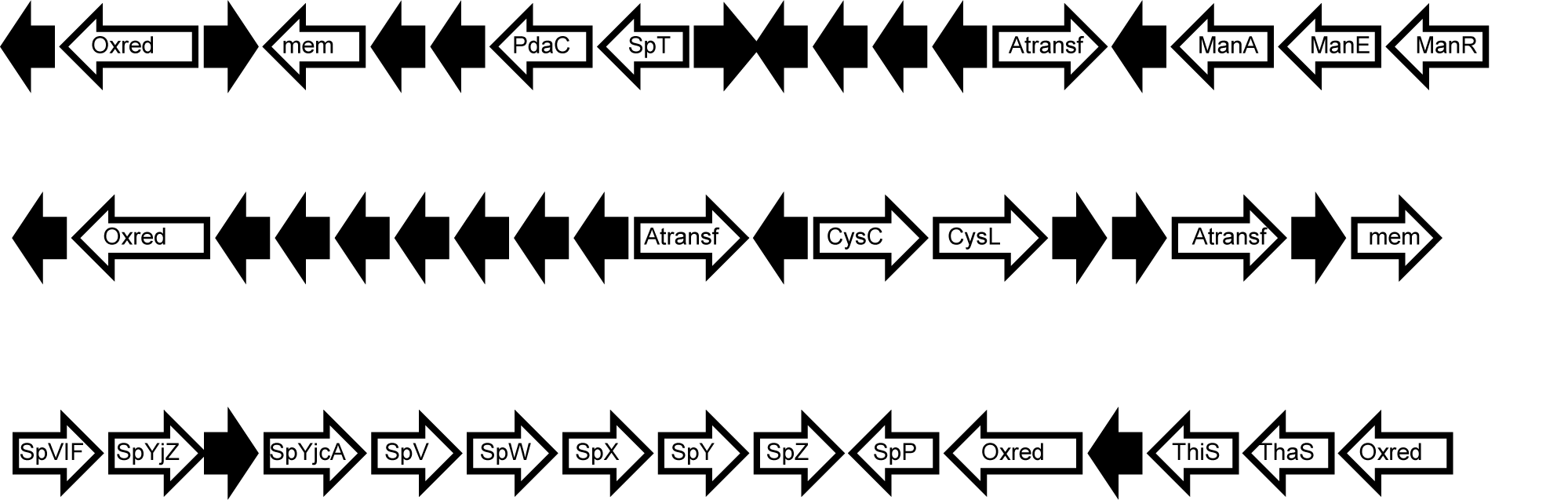

Supplement: Supplementary file 3 — 10.1186/s12934-016-0571-y Integrated B. subtilis DNA. Figure shows open reading frames of the high molecular weight DNA from B. subtilis 168 integrated into the E. coli K12 MG1655 chromosome. Hypotheticals with unknown function are highlighted black. Oxred: oxidoreductase; Atransf: Acetyl transferase; Cys: cystathionine metabolism; Man: mannose metabolism; Sp: sporulation; PdaC: peptidoglycan metabolism; ThiS, ThaS: thiamine metabolism. [file 12934_2016_571_MOESM3_ESM.tif]

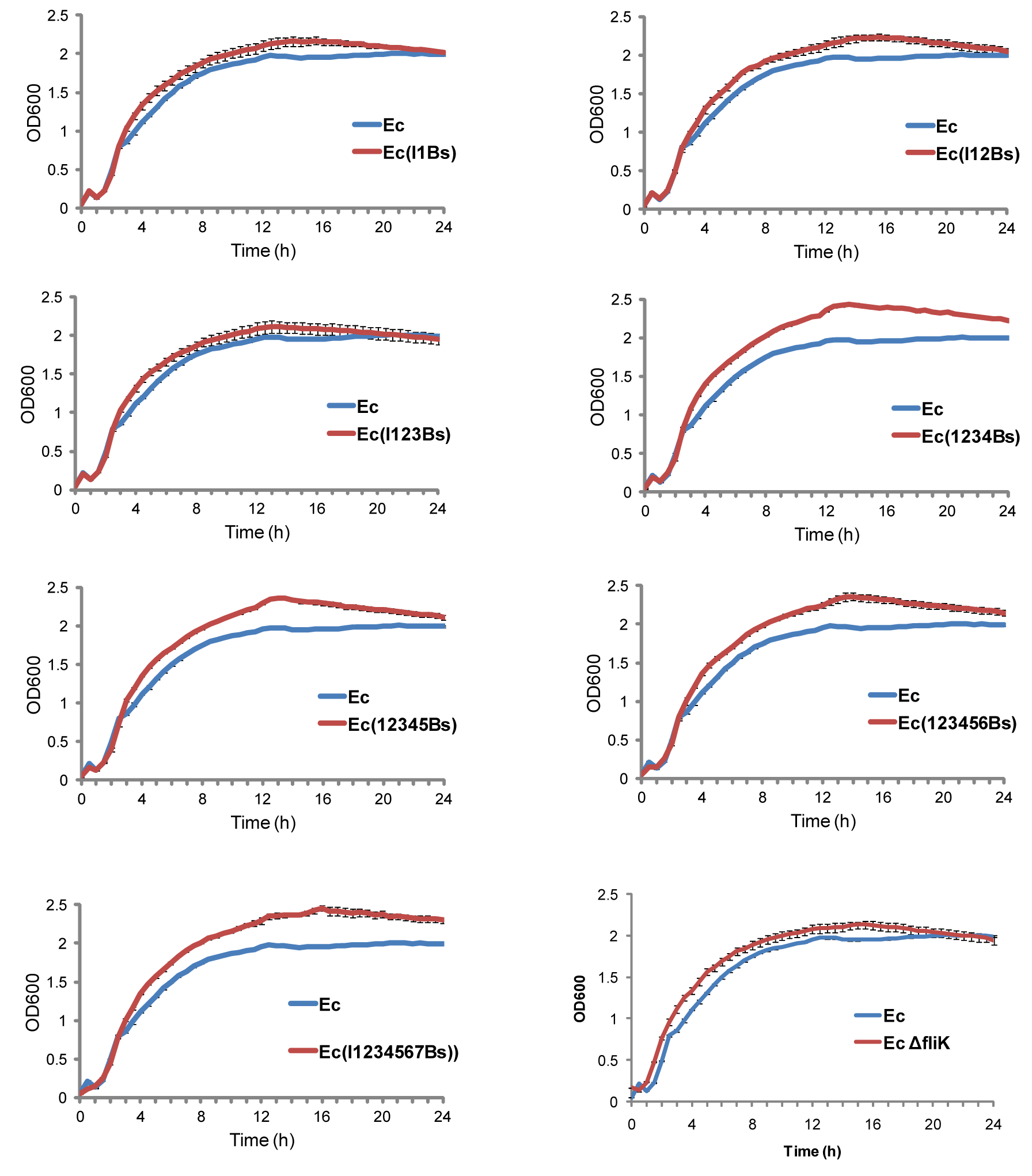

Supplement: Supplementary file 4 — 10.1186/s12934-016-0571-y Growth rates. Figure shows growth rates of the engineered strains Ec(I1Bs), Ec(I12Bs), Ec(I123Bs), Ec(I1234Bs), Ec(I12235Bs), Ec(I123456Bs), Ec(I1234567Bs) and strain EcΔfliK compared to E. coli K12 MG1655 wild type (Ec) measured with the microplate reader (Fluostar Omega). The means and standard errors were calculated from three biological replicates. The integration into the fliK locus did not have a negative effect on the growth of the host E. coli. [file 12934_2016_571_MOESM4_ESM.tif]
